# Reducing State Conflicts between Network Motifs Synergistically Enhances Cancer Drug Effects and Overcomes Adaptive Resistance

**DOI:** 10.3390/cancers16071337

**Published:** 2024-03-29

**Authors:** Yunseong Kim, Sea Rom Choi, Kwang-Hyun Cho

**Affiliations:** Laboratory for Systems Biology and Bio-Inspired Engineering, Department of Bio and Brain Engineering, Korea Advanced Institute of Science and Technology (KAIST), Daejeon 34141, Republic of Korea; verato@kaist.ac.kr (Y.K.); cgs@biorevert.com (S.R.C.)

**Keywords:** combinatorial drug targets, drug target discovery, network control, cell state transitions, systems biology

## Abstract

**Simple Summary:**

The heterogeneous response of cancer cells to targeted drugs is associated with the state transition dynamics of a molecular network. Identifying combinatorial drug targets to compensate for these heterogeneous responses can counteract adaptive resistance in cancer. To achieve this, we developed an algorithm called “merged transition map”, which explores essential state transition dynamics to identify combinatorial drug targets. Our analysis showed that drug-induced state conflicts within the molecular regulatory motifs of a network can result in heterogeneous responses. Moreover, we found that addressing these conflicts with additional perturbations can synergistically improve drug efficacy. Compared to other network control algorithms, our approach showed higher performance in drug efficacy of the suggested combinatorial target pairs with reduced computational complexity. Furthermore, by applying the MTM on a Boolean network model, we identified a new target combination that induces apoptosis in gastric cancer, supported by previous experimental data.

**Abstract:**

Inducing apoptosis in cancer cells is a primary goal in anti-cancer therapy, but curing cancer with a single drug is unattainable due to drug resistance. The complex molecular network in cancer cells causes heterogeneous responses to single-target drugs, thereby inducing an adaptive drug response. Here, we showed that targeted drug perturbations can trigger state conflicts between multi-stable motifs within a molecular regulatory network, resulting in heterogeneous drug responses. However, we revealed that properly regulating an interconnecting molecule between these motifs can synergistically minimize the heterogeneous responses and overcome drug resistance. We extracted the essential cellular response dynamics of the Boolean network driven by the target node perturbation and developed an algorithm to identify a synergistic combinatorial target that can reduce heterogeneous drug responses. We validated the proposed approach using exemplary network models and a gastric cancer model from a previous study by showing that the targets identified with our algorithm can better drive the networks to desired states than those with other control theories. Of note, our approach suggests a new synergistic pair of control targets that can increase cancer drug efficacy to overcome adaptive drug resistance.

## 1. Introduction

Despite advances in immunotherapy and surgical treatments of tumors, drug treatment remains a principal strategy against cancer [1]. The advantages of anti-cancer drug treatments lie in better and wider access to target cancer cells throughout the body with higher sensitivity, recovery rates, and relative cost-effectiveness [2]. Recent developments of anti-cancer drugs that specifically target mutated oncogenes or signaling molecules of a regulatory network in cancer cells not only compensates for causes of tumorigenesis but also complies with the principles of precision medicine [3]. Nevertheless, the recurring challenge is that cancer still cannot be treated with drug treatments, since it eventually acquires resistance through repetitive drug treatments [4].

Drug resistance is classified into three types: primary, acquired, and adaptive [5]. Primary resistance occurs through the impact of pre-existing mutations on the regulatory network that initiate tumorigenesis and block the effects of anti-cancer drugs. Acquired resistance emerges when cancer cells accumulate additional mutations, causing them to become unresponsive to treatment. Adaptive resistance, perhaps the most complex, arises from undesirably activated regulatory motifs such as feedback or crosstalk during the cancer cell state transition, which reduces drug efficacy [6,7]. Therefore, unlike primary and acquired resistance, it is necessary to address the dynamic mechanisms of cell state transitions after drug treatment beyond static information such as mutation profiles to overcome adaptive resistance [8,9].

With recent developments in systems biology and control engineering, understanding of dynamics of state transitions within various cellular networks have been greatly improved [10,11]. In particular, a Boolean modeling scheme recapitulates the switch-like behaviors of molecules within a regulatory network by representing a functional activity of each node in the network with only ON (1) or OFF (0) states [12]. The scheme can successfully implement the complex state transition dynamics of biological networks by using parameter-free logical equations [13]. The activity of marker molecules can define cellular phenotypes, often indicating cell viabilities and thus representing drug responses [14]. In a nominal state without any external disturbance, the cellular network state naturally transits towards a more stable state through a series of state transitions, which is known as a state transition path. As a result, all the states eventually converge to attractors, those of a few states with higher stabilities. These attractors can be represented as local valleys among an attractor landscape, which is the plane surface of possible network states [15]. The stability of each state on the landscape can be measured by the size of its basin of attraction, which defines the relative size of neighboring states that converge to an attractor [16]. With this, various studies on state control algorithms with Boolean networks have suggested probable molecular targets called “control targets” to regulate cellular phenotypes by pinning or permanently fixing the functional activity of each target node to ON (1, activation) or OFF (0, inhibition) [17]. However, these strategies utilize only the initial and final states of a network, which cannot recapitulate the drug response dynamics that trigger adaptive resistance due to dynamic stochasticity after drug treatments.

After anti-cancer drug administration of a target molecule in the network, its state changes gradually influence the state of other molecules in the network. The sequence of which molecules are randomly affected depends on dynamic stochasticity, such as expression levels, molecular activity, and probability of physiochemical interactions [18]. Despite a uniform regulatory mechanism of the molecular network, this results in drug influences that activate different signaling cascades in cancer cells and ultimately form their own stably activated regulatory motifs [19]. Molecular regulatory networks within a cell can be subdivided into interconnected strongly connected components (SCCs) called multi-stable motifs that have multiple stable states in their respective attractors [20]. However, as some of these stable states are biologically undesirable, drug-treated cancer cells consist of undesirably activated multi-stable motifs that then can inhibit desirably activated regulatory motifs that increase drug efficacy. Thus, a population of single-drug-treated cancer cells can heterogeneously be stabilized, and some of them may enter a drug-resistant state. These diverse responses cause adaptive resistance to the anti-cancer drug, which requires additional drug treatments in combination to compensate for the heterogeneous responses. Hence, the question arises as to whether we can induce desirable homogeneous responses in cellular systems by suggesting an optimal combination target for the given anti-cancer drug treatment.

To answer this question, we have developed a new combinatorial target search algorithm called “merged transition map” (MTM). The MTM considers essential network dynamics for identifying combinatorial control targets that reduce heterogeneous responses of a given anti-cancer drug by extracting transition paths between initial and desired network states. We applied the MTM to a gastric cancer model [21] to validate and test the performance of our strategy. By extracting essential state transition dynamics induced by target node perturbations, we revealed that competitive stabilization between multi-stable motifs caused by the dynamic stochasticity of drug effects spreads throughout the network and ultimately increases the heterogeneity of drug responses. Moreover, we found that reducing dynamic stochasticity of the molecules with frequent activity changes during the drug-induced state transitions can reduce competitive stabilization of multi-stable motifs. In addition, this can also synergistically increase the efficacy of the given targeted drug. We also discovered that conflicts between multi-stable motifs can steer the network into undesired states that can cause adaptive resistance. Thus, the suggested MTM combinatorial target can overcome resistance by regulating the nodes interconnecting these motifs. The MTM also identifies the nodes that frequently flip the most after a specific drug perturbation, positioning them as optimal combination targets for the given drug. Our findings underscore the possibility of the MTM identifying synergistic targets to counteract adaptive resistance by diminishing heterogeneous drug responses. Altogether, our study provides new insights into enhancement of a biological systems approach that can open a new paradigm in identifying combinatorial targets to compensate adaptive drug resistance in cancers.

## 2. Results

### 2.1. Controlling Frequently State-Flipping Nodes Reduces Heterogeneous Drug Responses

**Figure 1:**
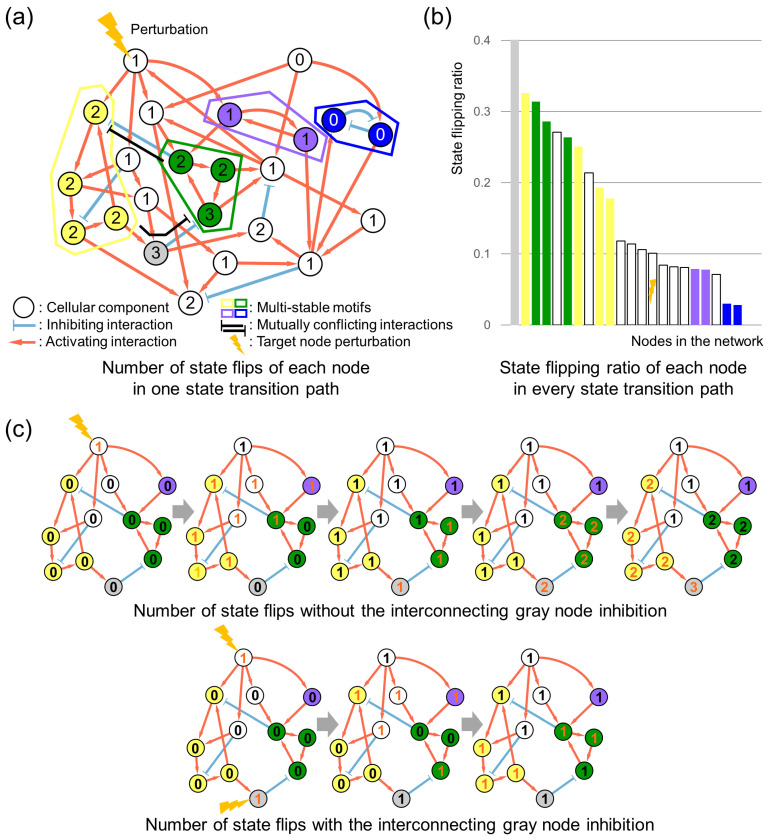
Conflicting interactions between multi-stable motifs increases node state flips after perturbation. (**a**) Four multi-stable motifs, colored yellow, green, purple, and blue, have different topological locations, with the given perturbation marked with an orange lightning. The yellow and green motifs are mutually inhibiting each other and the interconnecting node in between, which are both regulated directly by the perturbation node. The purple motif was directly regulated by the perturbation node, but did not interact with other motifs. The blue motif was not regulated by the perturbed node. The gray node interconnects yellow and green multi-stable motifs with mutual inhibitory relationships. For the simplified representations, the number of state flips marked on each node accordingly after the given node perturbation from the upper-left node perturbation is measured from a randomly selected path in every possible state transition from a single nominal network attractor to a single regulated network attractor. (**b**) The bar graph shows the weighted state-flipping frequencies of each node in the MTM with their respective colors and a perturbation mark. (**c**) Partial networks of the yellow and green motifs and their interconnecting gray nodes show their gradual state flips from the node perturbation to an attractor. Newly state-flipped nodes are numbered in orange.

To topologically illustrate which node in a regulatory network flips the most after perturbation, we measured the number of state flips of each network component based on its relative localization within the entire network as shown in Figure 1a. We analytically represented this by using an asynchronous Boolean model that is simplified but still effectively encapsulates essential network topologies of the model (see Supplementary Figure S1 for network logic equations and identified attractors). The relative topologically positioned locations of each node, associated with the given target that is perturbed, can be explored by measuring state-flipping frequencies of each node. Once the target is perturbed, its effect competitively disseminates throughout the network and causes state conflicts with the other nodes. These affected nodes ultimately flip their states until the entire network states converge to three possible attractors of the target node-perturbed network. Through that state transition path, the number of state flips from individual nodes may vary based on their topological locations. Notably, the yellow and green multi-stable motifs are both directly regulated by the perturbation node and exhibit mutual inhibition mediated by the interconnecting node located between them. Note that the nodes from the yellow and green motifs experience more state flips than the other nodes after the perturbation, indicating that a number of state flips increases when the state conflict between the motifs is more frequent. Moreover, the nodes located in between the conflicting motifs tend to flip the state of nodes even more frequently. Thus, the interconnecting nodes between the multi-stable motifs are intrinsically associated with state conflicts between these motifs and flip more frequently after the target node perturbation.

Figure 1b shows an average state-flipping ratio of each node, which defines a number of states flipped from each node divided by the number of state transitions, throughout every state transition path to one of three possible attractors after the target node perturbation. These results further support the induced flipping frequency of the interconnecting node during the competitive stabilization. Figure 1c shows that blocking the interconnection between the motifs ultimately can reduce competitive stabilization by properly pinning the frequently flipping node state according to the desired phenotype (see Section 4 for details). Therefore, identifying the most frequently flipping node and properly controlling the node in combination with the given target will enhance its effect by blocking the state conflicts between the multi-stable motifs and reducing competitive stabilizations responsible for heterogeneous responses.

### 2.2. MTM-Based Weighted Flipping Frequency Calculation Enables Synergistic Target Identifications

**Figure 2:**
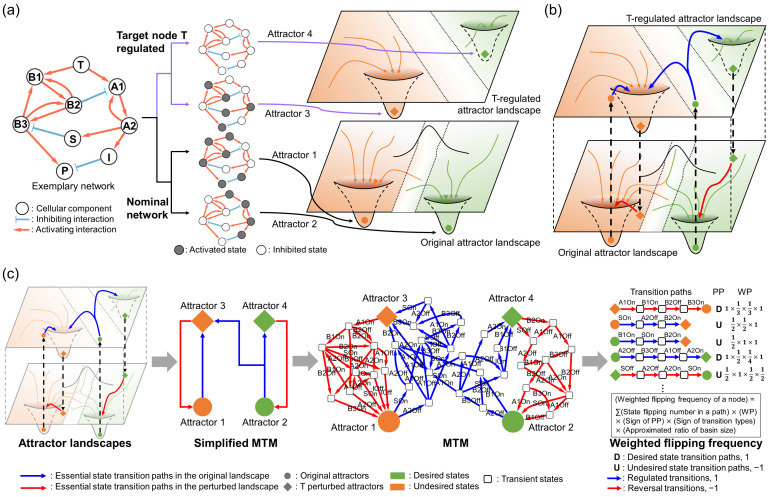
The MTM can identify most frequently flipping node after the target node perturbation. (**a**) From an exemplary network with a target node T, we can calculate two attractor landscapes, one with nominal or T-regulated network, with a set of attractors identified respective to each landscape. (**b**) When a target node is perturbed, the original landscape is transformed into a perturbed landscape, where an initial attractor state may no longer be a stable attractor anymore after the perturbation. The perturbed attractor landscape of the network can return to the original landscape once the target is unpinned and converges into a new attractor state with higher stability. These landscapes can be merged to form an MTM. (**c**) Schematic workflow of the MTM. Essential state transition paths in the original or perturbed landscape are represented by blue or red arrows, respectively. From the MTM, phenotypic preferences are color-coded from orange to green to represent undesired to desired states. Transient states are in empty squares. The most frequently flipping node can be identified with weighted flipping frequencies of each node by multiplying weighted probability (WP), sign of transition types, phenotypic preferences (PP), approximated ratio of basin of attraction of the initial attractor over the whole attractor landscape, and the number of state flips of each node within state transition paths.

To systematically explore the dynamic repertoires after a target node perturbation within a network, we utilized the MTM to extract the essential dynamics of node state changes induced by the perturbation. Without external disturbances, a molecular network resides in a few highly stable attractor states, forming an original attractor landscape. When a perturbation, such as to molecule T, is introduced, it alters the molecular interactions with its adjacent molecules, leading to the deformation of the original attractor landscape into the T-regulated attractor landscape. This change destabilizes the original steady states of the network, initiating state transitions to new stable attractor states through various state transition paths. The altered node state of T progressively affects the other network components, causing continuous flips of their states. Eventually, this results in the network stabilizing into a new attractor state within the T-regulated landscape (Figure 2a). On the other hand, the T-regulated attractor landscape of the network can return to its original landscape once the target is unpinned (e.g., a decrease in the effects of the drug treatment through degradation, excretion, or a conditional gene-knockout system.). As a result, the network returns to its original attractor landscape and converges into a new attractor state with a higher stability.

Figure 2b shows that these landscapes comprising essential dynamic information can be merged to form an MTM, which then can be used to identify a synergistic target pair to the given control target by comparing their flipping frequencies. Biologically, the molecular network remains in a stable attractor state in the absence of external influences like drug perturbations. Upon perturbation, the network state can only transit from attractors in the original landscape to those in the regulated landscape. Therefore, the MTM captures all potential state transition paths triggered by the perturbations. Consequently, we can drive the states of drug responses towards desired states by extracting key state transitions from the MTM and developing a control strategy for them. A schematic representation of how the MTM can extract the essential dynamics of the network state transitions is shown in Supplementary Figure S2.

We calculated desired state transition paths by utilizing the weighted flipping frequencies of each node from the MTM within an exemplary Boolean network, as shown in Figure 2c (see Supplementary Figure S3 for network logic equations and identified attractors). These state transition paths can be categorized based on the relative phenotypic preferences of each attractor into two types, desired (D) or undesired (U), according to the relative phenotypic preferences of the attractors at both ends. Specifically, a state transition path initiated from a relatively undesired attractor to a desired attractor is termed a “desired path”. Conversely, a state transition path initiated from a relatively desired attractor to an undesired attractor is termed an “undesired path”.

To identify the optimal target, we explored the most frequently flipping node from the undesired paths by comparing weighted flipping frequencies of each node within the network. MTMs can be factorized into a series of consecutive state transition paths, either D or U. The significance of each transition path from the overall drug response dynamics was considered by multiplying the weighted parameter proportional by the ratio of the basin of attraction from each initial attractor for the corresponding transition path. We quantitatively identified the optimal target node with the highest weighted flipping frequency as a synergistic target pair to the given target node by multiplying the weighted probability of each state transition, the number of state flips in each path, significance value of each transition path, and their transition types (see Section 4 for details). Stabilizing the identified node, which shows the most frequent state flips in undesired paths, into a desired state can eliminate these unwanted state transitions, thereby enhancing drug efficacy. As a result, the MTM successfully identified node S from the exemplary network as the most synergistic combinatorial target of the given node T, which is validated by computational simulations of synergistic effects in every possible two-node combination (see Supplementary Figure S4 for an overall workflow chart).

### 2.3. MTM Suggested Synergistic Pairs That Are in Well Accord with Prior Knowledge

**Figure 3:**
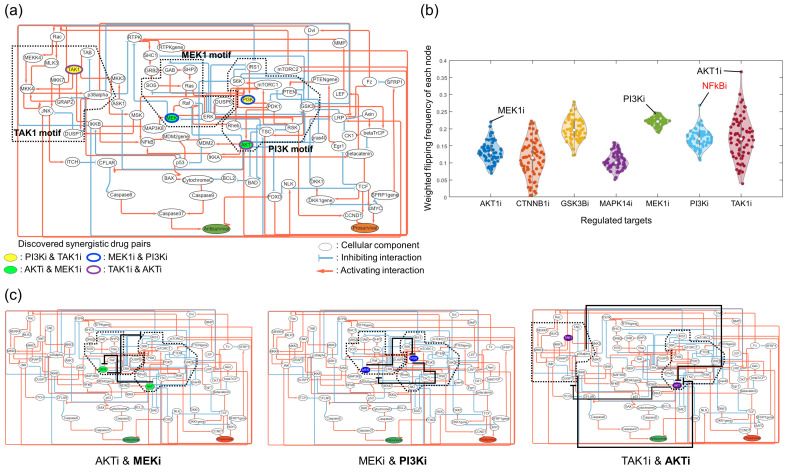
Identification of novel synergistic target pairs using the MTM. (**a**) The logical Boolean model of gastric cancer has two phenotypic nodes, antisurvival and prosurvival. The desired phenotypic node, antisurvival, is colored green, while the undesired phenotypic node, prosurvival, is colored orange. Four of the identified synergistic target pairs are filled or bordered according to the same color. Three of the identified multi-stable motifs related to the synergistic pairs, TAK1, MEK1, and PI3K, are boxed in black dots. (**b**) The MTM suggested synergistic targets that are well in accord with the previously identified pairs. Weighted state-flipping frequencies of every node in each of the targets are plotted. (**c**) Each synergistic pair suggested by the MTM shows a similar mechanism: one target regulates multi-stable motifs to drive desired states, while the other target blocks the negative interactions between the motifs. The targets blocking the negative interactions are in bold letters. The negative interactions blocked by the targets are depicted with solid black links.

We applied the MTM to a logical model of gastric cancer cells to validate that our approach can identify synergistic target pairs that are well in accord with previous studies. Flobak et al. constructed a gastric cancer cell logical model with 75 nodes and 149 links, discovered synergistic drug pairs from their model (Figure 3a), and experimentally validated them with the AGS gastric cancer cell line [21]. Their identified synergistic drug pairs were PI3Ki and TAK1i, MEK1i and PI3Ki, AKTi and MEK1i, and TAK1i and AKTi, which consisted of three multi-stable motifs in the network. By applying the MTM in the model, we identified the three synergistic pairs—AKTi and MEK1i, MEKi and PI3Ki, and TAK1i and AKTi—that were consistent with their results in addition to our novel target pair with supporting evidence [22,23]: PI3Ki and NFkBi (Figure 3b). These results showed that the MTM can successfully predict synergistic effects of the gastric cancer model that are concordant with previous studies. In summary, the MTM is able to recapitulate the essential drug response mechanisms within an experimentally validated model.

Figure 3c shows that the synergistic target pairs identified by the MTM are topologically aligned with the targets determined using multi-stable motifs and their interconnecting nodes. It shows that the given regulatory targets, including AKTi, MEKi, and TAK1i, regulate their multi-stable motifs, while their corresponding pairs—MEKi, PI3Ki, and AKTi, respectively—are their inhibitory interconnecting nodes. Thus, the MTM not only successfully identified synergistic target pairs that are consistent with previous studies but also interpreted the synergistic mechanism of these pairs with structural information of the network.

### 2.4. MTM Reveals Novel Synergistic Pairs That Differ from Other Control Theories

**Figure 4:**
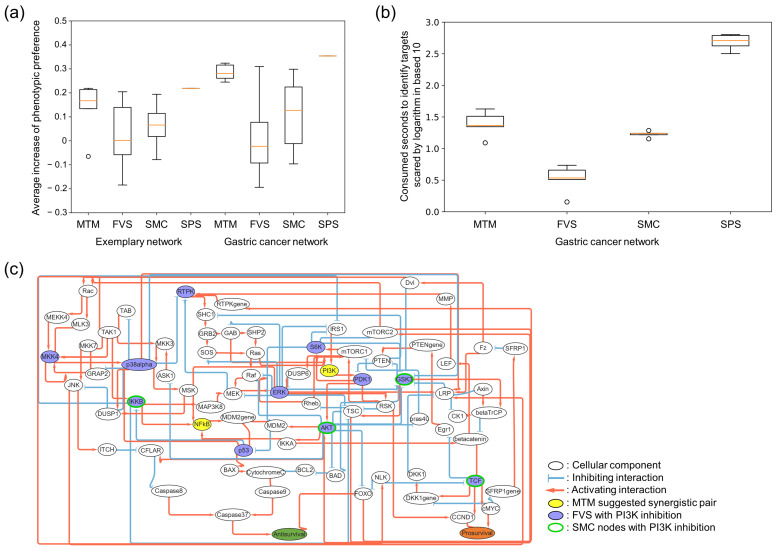
Comparing performance of the MTM and other control theories. (**a**) The graph compares control effectiveness and the increase in average phenotypic preference of attractor states after regulating the identified target pair suggested by the MTM and the other control theories. In the case of the MTM, five synergistic targets with the highest weighted flipping frequencies were selected. The left side of the graph shows the control effectiveness in controlling the exemplary network in Figure 2b. The right side of the graph shows the control effectiveness in controlling the gastric cancer network in Figure 3a. (**b**) The graph compares the time consumed for the target identification using MTM, FVS, SMC, and SPS strategies on the gastric cancer network model. Each algorithm is iteratively performed five times. (**c**) The novel synergistic target pair identified by the MTM in the gastric cancer model, PI3Ki and NFkBi, is colored yellow. The suggested control targets by FVS and SMC are colored purple and green, respectively.

Due to the absence of established algorithms for identifying optimal synergistic targets for a given drug, we compared the control effectiveness of the MTM with other general target search algorithms. For this, we implemented feedback vertex set (FVS) [24], stable motif control (SMC) [25], and systematic perturbation simulation (SPS) [26] to identify synergistic target pairs using these strategies accordingly. These algorithms utilize different approaches to search for network control targets with the given network model: (1) FVS uses structural information of a network to disconnect all feedback loops and identifies control targets of the resulting tree-like network structure, (2) SMC decomposes a network into various motifs with dynamic information that can be used to identify control targets from a succession diagram of attractors, and (3) SPS calculates every possible state transition to identify the most effective synergistic targets by manually pinning the states of every possible two-node pair, though with high computational complexity. To quantitatively analyze the control effect of each method, we applied them in the same models and measured their relative changes in phenotypic preference in averaged network states after regulating those targets accordingly (Figure 4). For FVS and SMC, which proposed numerous control target sets to exactly control the network into a desired state, we computed the average control effectiveness of every possible two-node pair combinations from the suggested sets. We then compared these results with the target pairs suggested by the MTM to determine the relative effectiveness of each. A detailed explanation of calculating control effectiveness is provided in the Section 4.

We showed that the average control effects of targets suggested by the MTM are more substantial than the other corresponding models (Figure 4a). We also showed that our results using the MTM can identify synergistic targets and that their control effects are comparable to those using the SPS strategy with the most effective target sets. Moreover, we presented that the average time consumption during computational simulations using the MTM was much less than that of using the SPS (Figure 4b). Finally, we further presented that the MTM can identify novel synergistic target pairs that were not identified using the FVS or SMC (Figure 4c). These results indicate that the control target identification using the MTM can identify more optimal and novel synergistic targets than those of the other algorithms in terms of both control effectiveness and computational complexity.

## 3. Discussion

It has long been suggested that evolutionary gains in network complexity in a cell are not merely incidental but the results from adaptive capacities that reinforce information processing [27]. Our results indicate that the conflicts between multi-stable motifs cause heterogeneous responses upon a perturbation, which may not be beneficial to cells. Such varied responses may be detrimental to the cells, which may ultimately become diminished through natural selection, since uniform responses might be more efficient in optimizing their responses to external stimuli. Yet intriguingly, these conflicting structures between multi-stable motifs are more prevalent in biological networks than in random networks (see Supplementary Figure S7 for further details). These structures commonly appear across various gene sets, suggesting that the conflicting dynamics induced by positive feedback loops are a prevalent feature of cellular networks selected over evolutionary time [28]. Cancers, being evolutionarily flexible, often exhibit heterogeneous responses to single drug treatments due to their possession of diverse cancer hallmarks, providing survival advantages [29]. This suggests that conflicting dynamics between multi-stable motifs in fluctuating environments may confer survival benefits, but also complicate achieving desired cell states with single-drug treatment [30]. Thus, understanding how these multi-stable motif structures are mutationally enriched in cancers and dynamically affect each other is crucial for overcoming adaptive drug resistance in cancers. The MTM suggests combinatorial targets based on essential network dynamics independently of tissue contexts and holds promise for identifying synergistic drugs across cancer types.

The general control target search algorithms focus on modulating every node state within a network converging to a desired state. This approach often necessitates the perturbation of a substantial number of target nodes for proper network controls. However, to control the phenotype of a cancer cell to a specific desired state, it is not imperative to regulate every node within the network. Instead, modulating a few marker nodes that determine the phenotype is sufficient. Moreover, simultaneously controlling a large number of target nodes is nearly infeasible. These biological aspects underscore that effective control strategy of biological networks should prioritize control of phenotypic marker nodes through a realistic number of target nodes. In our study, we focused on the biological feasibility of the control targets identified. Our approach is based on two key considerations. First, the challenge in cancer therapy is not the identification of new drug target genes, but rather addressing the prevalent issue of drug resistance in well-established target drugs. To this end, the MTM algorithm pinpoints the most synergistic drug targets within given perturbations of target nodes. Second, given the impracticality of controlling multiple genes or biological molecules within a cell, we limited the number of control targets to two. As a result, the MTM can show practical applicability on target identifications to overcome drug resistance in cancers, enhanced over prior control theories.

Several motif-based network control theories exist, and identifying targets regulating each of these motifs within a network is crucial to govern the entire network [31]. In addition, numerous system biological strategies have been developed to measure phenotypic changes according to modified expressions of a gene set [32]. Together with constructed Boolean models of cancer, in silico simulations, which implement those control theories that aid in predicting drug responses and interpret molecular mechanisms, have been widely used in various targeted cancer therapies [33]. A common way to regulate the entire gene set is by identifying their master regulator, a gene that regulates certain gene sets, as a control target [34]. However, heterogeneous responses of the gene sets and multi-stable motifs can dramatically reduce the control effects. Our results have revealed that these conflicts between the motifs, rather than the efficacy of a certain control target, give rise to the heterogeneous drug responses of the given anti-cancer drug. Thus, our MTM-based network control strategy can identify novel and effective synergistic pairs to common pharmacological targets that can reduce heterogeneous drug responses.

Experimental validations on control targets identified by analyzing cellular network models are crucial for substantiating in silico studies such as the MTM. However, measuring the dynamics of the mutual inhibitions between feedback loops proposed in this study requires extensive labor and resources. Therefore, instead of in-house experiments, we opted for an indirect validation method. We assessed whether the MTM identifies combinatorial targets mentioned in previous research utilizing two network models with extensive experimental validations [21,35]. Additionally, we referenced supporting experimental publications for a novel combination not covered in the studies [22,23]. While these studies may not be fully oriented to reproducing the mutual state conflict between positive feedback loops, the consistency between the MTM analysis results and actual drug responsiveness of cancer cell lines suggest their validity. Uncertainties in this study can be addressed through further analysis of various cancer cell models recapitulating network dynamics with a focus on positive feedback loops and supplementing time-series experimental data on network dynamics associated with drug response [36]. In clinical practice, despite the identification of major cancer-related genes and the development of targeted drugs for each mutation, adaptive resistance remains as a challenge with single-drug treatment. The MTM, initially designed to identify combination targets that minimize adaptive resistance for specific anticancer drugs, leverages the inherent drug response dynamics of cancer cell networks with minimal intervention. This approach can mitigate drug overuse, which can lead to toxicity issues, making MTM highly applicable in clinical settings [37].

In this study, we applied the MTM specifically to single-node perturbations induced by small-molecule inhibitors. However, it is important to note that many cancer treatments such as lipid nanoparticles or siRNAs/mRNAs typically influence multiple molecular targets at once. This results in more significant changes in network states than what is observed with small-molecule treatments. Despite the complexity introduced by treatments that simultaneously affect various molecules within a network, the MTM remains applicable to identify the most synergistic target pair reducing heterogeneous responses. The key to its applicability lies in the fact that regardless of how many molecules are affected by the perturbation, the attractor landscape of the cancer cell network undergoes a single but significant change due to the treatment. This change prompts state transitions within the network, all of which can be effectively analyzed using the MTM.

In biological networks, heterogeneous cellular responses to external stimuli are commonplace. Our MTM-based target identification approach, which reduces competitive stabilization, offers a robust method for analyzing network dynamics. The heterogeneous cellular responses common in biological networks to external signals make our MTM-based target identification strategy versatile for analyzing network dynamics. This approach is effective across various network models, whether deterministic or stochastic. This is because iterative simulations with numerous random initial states can bring randomness of the stochastic nature of biological networks to synchronous Boolean or ordinary differential equation (ODE) networks with deterministic logics. For instance, we have shown that extracting the MTMs and identifying synergistic targets based on state-flipping frequencies are also effectively applicable in synchronous Boolean network models (see Supplementary Figure S5 for details) [35]. Thus, competitive stabilization of a network state is not merely an artifact of stochastic or asynchronous updating of logic of Boolean models.

Two questions arise from our findings. First, two of the suggested targets by the MTM may not be interchangeable, even though it suggests combinatorial synergistic targets of a given target drug. In addition, the optimal synergistic pair of a specific target suggested by the MTM is designated to the given target and can be changed when the identified synergistic target is reversely assigned as a new given target. However, it is comprehensive, since synergistic target pairs in cancer therapies often play different roles in regulating cell states [38]. Second, some of the synergistic pairs for a certain drug target, which is located relatively lower in a hierarchy from the network, cannot be identified. However, this aligns with biological expectations, since common molecules targeted by cancer drugs generally reside at higher hierarchies in the network to regulate a wide range of downstream molecules [39] (see Supplementary Figure S6 for further details). Therefore, our strategy of identifying synergistic targets using the MTM is generally applicable to any modeling scheme without compromising the biological insights for cancer therapeutics.

## 4. Materials and Methods

### 4.1. Boolean Network Models and Simulation Schemes

We employed Boolean models with asynchronously updates. In this framework, variables can only be 1 or 0 to represent the activity of the corresponding biological component as “active” or “inactive”, respectively. Each Boolean network component and its relationship with other nodes can be represented using Boolean operators AND, OR, and NOT. The logical regulatory equation assigned to each component updates the state of corresponding components and causes the state flips between 1 and 0. During each simulation step, one of the variables is randomly selected and updated according to its logical regulatory equation. The stochastic spreading of serial molecular state flips through the network is implemented in Boolean modeling by asynchronously updating a state of randomly selected single node for each step of network state change. In the case of the gastric cancer model [21], we followed multileveled variables (named prosurvival, antisurvival, Caspase3/7, and CCND1) and their corresponding logical formulae.

### 4.2. Identification of Every Possible Attractor

To identify every possible attractor in a Boolean network, we implemented the algorithm suggested by He et al. This algorithm identifies attractors from a simplified Boolean network by determining constant nodes according to the network logic, perturbation inputs and initial states [40]. We hard-coded such an algorithm with MATLAB R2021a. All the identified attractors were validated by verifying whether they remained within the identified states during network simulations.

### 4.3. Calculation of Phenotypic Preference for Each Attractor

To determine the phenotypic preference of each attractor, we first established the phenotypic marker nodes and desirable network states of each model. For attractors that remain within a single state, the corresponding state value of the marker node directly represents the phenotype of that attractor. Conversely, for those with multiple states, such as a complex attractor in an asynchronously updating Boolean modeling scheme, the phenotype is represented by the average state value of marker nodes across these states. In case of the gastric cancer model suggested by Flobak et al. [21], we adhered to the phenotypic outputs delineated by the authors. We also defined that the state of the antisurvival node has a higher value than that of the prosurvival node as our desirable attractor.

### 4.4. Calculation of State Transition and MTM Extraction

To induce the state transitions of network models, we pinned the corresponding target node state as 1 (ON) or 0 (OFF), ensuring their states remained fixed during the simulations. Then, we iteratively simulated the state transitions by using every possible attractor of the model before and after the pinning and target perturbation as initial and final states, respectively. Every state transition from the initial states to reachable attractors from the perturbed network is then merged into a MTM.

### 4.5. Calculation of State-Flipping Frequency for Each Node in the MTM

The state-flipping frequency of each node within an MTM is calculated by following three steps. First, state transition probabilities of each transition within the MTM are calculated by assuming that state transition probabilities for consecutive states are evenly distributed in every branch. Second, the MTM is factorized to state transition paths, each anchored by attractors at both ends. Phenotypic preferences of each path are assigned by comparing relative preferences of the both attractors. Third, we calculate the weighted flipping frequency of each node by adding every multiple of state-flipping numbers from the state transition probability of the path and the phenotypic preference, as well as state transition types. The weighted probability of each state transition path is calculated by multiplying the factor of corresponding transient states according to their outgoing edges. The phenotypic preferences are then multiplied by 1 if the path is desirable or −1 if undesirable. A factor of 1 is multiplied in the state transition types if the transition is included within the regulated attractor landscape (regulated transitions), or a factor of −1 is multiplied if the transition is included within the original attractor landscape (reversal transitions).

### 4.6. Estimating Significance of Each Transition Path Using Approximated Basins of Attraction

Due to the computational complexity of calculating the basin size of each attractor in an asynchronously updated Boolean model, we employed an approximation method. We estimated the basin of each attractor by tracing a number of converged attractors from a pool of sampled initial states. To sample the initial states, we randomly choose the state of each node in the network between 1 and 0 in uniform distribution. We choose 10,000 non-overlapping initial states and trace converged attractors from each initial state to approximate the basin size of each attractor. To calculate the significance of a specific transition path, the ratio for the number of converged initial states to the starting attractor within the path over 10,000 initial states is multiplied by the weighted flipping frequency of that path.

### 4.7. Identification of Multi-Stable Motifs and Interconnecting Nodes

After calculating every possible attractor from the original attractor landscape as well as every reachable attractor from these attractors once regulated, we identified network motifs, which are SCCs, that possess at least two distinct stable states in attractors. The existence and a parametric region of multi-stability from the identified network motifs is then measured with BioSwitch, developed by Yordanov et al. [41], which computes bifurcation diagrams of network motifs from the limit points. The interconnecting nodes between the multi-stable motifs are identified by the PathLinker algorithm, which connects source nodes to target nodes on a given network structure by calculating k-shortest paths, developed by Gil et al. [42].

### 4.8. Determination of Control Type for the MTM Identified Synergistic Targets

To hinder serial state flip propagation between multi-stable motifs, a proper control type of ON or OFF state should be selected for pinning the synergistic target identified by the MTM. If the control effect of the given target node affects the identified synergistic target by turning it OFF, it has to be pinned to ON state for interfering its propagation, and vice versa. According to the control type of the given target node and the signs of connected interlinks between motifs and the identified target, the control effect of the given perturbation propagated to the synergistic target node is calculated. For this, we perform signal flow analysis to estimate signal propagation after perturbation using only topological information of the network [43]. As a tendency for an altered state of the identified synergistic target to be ON or OFF is calculated based on the given control node using signal flow analysis, the control type of the synergistic target can be determined as the opposite of this state.

### 4.9. Identification of Synergistic Target Pairs Using Network Control Theories

We implemented the original algorithms of FVS [24] and SMC [25] using hard-coded codes of Python 3.7.6. To compare the control effectiveness of the known control target search algorithm with the MTM, we limited the number of control target nodes to two. This adaptation was necessary because original algorithms often suggest controlling more than two nodes. We evaluated the control efficacy of every possible two-node pair selected from these suggested target lists and calculated their average control effectiveness. For instance, if a strategy proposes controlling nodes A, B, and C, we assessed the control effect on phenotypic nodes when controlling A and B, B and C, and C and A, respectively, to derive an average efficacy for two-node controls. To calculate average control effectiveness of target nodes suggested by the FVS, every possible two-node pair in the suggested target nodes is regulated and their corresponding phenotypic changes of attractors are measured. To calculate the average control effectiveness of two-node pairs suggested by the SMC, every succession path to desired steady states in a succession diagram is selected. Then, every possible two-node pair suggested from those succession paths is regulated and their corresponding phenotypic changes of attractors are measured. In the case of the MTM, five synergistic targets with the highest weighted flipping frequencies were selected.

### 4.10. Curation of Biological Network Structure from the OmniPath Database

To extract the biological network structures, we downloaded the OmniPath network structural data file from the archives (https://archive.omnipathdb.org/ (accessed on 28 March 2024)) [44]. The downloaded versions of two interaction data files were uploaded on 14 June 2018, and 26 April 2019. Those two data files were merged, and interactions without signs or directions were removed. Then, the interactions were cross-validated by leaving the links with confidence level A of the DoRothEA signed target gene–transcription factor interaction data [45]. Finally, the network with the largest number of connected components, with 3877 nodes and 10,814 links, was selected as a curated OmniPath network structure.

### 4.11. Randomization of Network Structure While Preserving Degree Distributions

To randomize link connections between the nodes from the curated OmniPath network structure, we first selected two non-overlapping nodes in pair and their outgoing links, as well as their corresponding target node. We then swapped these target nodes from each pair for a sufficient number of iterations. We discretely increased the number of iterations from 10% to 50% of a total number of links in the network by 10% increments for repeatedly generating randomized network structures in groups of 10. As a result, 50 randomized network structures were generated.

### 4.12. Extraction of Multi-Stable Motif Structures from the Curated OmniPath Network

To extract the multi-stable motifs with specific network structures from the curated OmniPath network and its randomized networks, we implemented the “pattern join” method suggested by Patra et al. [46], which iteratively screens the existence of a specific motif structure by joining smaller motifs within a network. The numbers of specific multi-stable motif structures overlapped from the given network were systematically explored by hard-coded MATLAB R2021a simulations.

## 5. Conclusions

Based on our results, we suggest that development of a control theory to reduce the heterogeneous responses on given perturbations by utilizing network dynamics is imperative to identify synergistic combinatorial targets to overcome adaptive resistance. We highlight the critical role of state conflicts emerging between multi-stable motifs induced by targeted drug perturbations on triggering adaptive drug resistance in targeted cancer therapies. Our study focuses on a common network structure featuring two mutually inhibitory multi-stable motifs, a configuration that leads to state conflicts and consequently heterogeneous drug responses and adaptive resistance. Notably, we revealed that combinatorial control of the interconnecting node with the given perturbation can resolve the state conflicts between the motifs. This induces the entirety of the states in a cancer network to a desired state by reducing heterogeneous drug responses. Since state conflicts occur only during transitions followed by perturbation, analyzing essential network dynamics and the state-flipping frequencies of each molecule in the transition paths between attractors is crucial for identifying efficient synergistic combinatorial targets.

## Data Availability

Dataset available on request from the authors.

## Supplementary Materials

The following supporting information can be downloaded at https://www.mdpi.com/article/10.3390/cancers16071337/s1. Figure S1: The logic equations of exemplary network models; Figure S2: The schematic representation of MTM and random stabilizations; Figure S3. The logic equations of exemplary network models; Figure S4. An overall workflow chart of the MTM based synergistic target identification; Figure S5. Identification of previously reported synergistic target pairs of the synchronously updated Boolean network model through MTM; Figure S6. Topological characteristics of synergistic pairs suggested by the MTM; Figure S7. Multi-stable motifs and their mutual inhibitions are common in biological network structure.

## References

[1] Falzone L., Salomone S., Libra M. (2018). Evolution of cancer pharmacological treatments at the turn of the third millennium. Front. Pharmacol..

[2] Debela D.T., Muzazu S.G., Heraro K.D., Ndalama M.T., Mesele B.W., Haile D.C., Kitui S.K., Manyazewal T. (2021). New approaches and procedures for cancer treatment: Current perspectives. SAGE Open Med..

[3] Ashley E.A. (2016). Towards precision medicine. Nat. Rev. Genet..

[4] Housman G., Byler S., Heerboth S., Lapinska K., Longacre M., Snyder N., Sarkar S. (2014). Drug resistance in cancer: An overview. Cancers.

[5] Sharma P., Hu-Lieskovan S., Wargo J.A., Ribas A. (2017). Primary, adaptive, and acquired resistance to cancer immunotherapy. Cell.

[6] Lee H.-S., Hwang C.Y., Shin S.-Y., Kwon K.-S., Cho K.-H. (2014). MLK3 is part of a feedback mechanism that regulates different cellular responses to reactive oxygen species. Sci. Signal..

[7] Chandarlapaty S. (2012). Negative Feedback and Adaptive Resistance to the Targeted Therapy of CancerAdaptive Resistance to Targeted Therapy in Cancer. Cancer Discov..

[8] Labrie M., Brugge J.S., Mills G.B., Zervantonakis I.K. (2022). Therapy resistance: Opportunities created by adaptive responses to targeted therapies in cancer. Nat. Rev. Cancer.

[9] Park S.G., Lee T., Kang H.Y., Park K., Cho K.-H., Jung G. (2006). The influence of the signal dynamics of activated form of IKK on NF-κB and anti-apoptotic gene expressions: A systems biology approach. FEBS Lett..

[10] Wolkenhauer O., Wellstead P., Cho K.-H., Sreenath S.N. (2008). Modelling the dynamics of signalling pathways. Essays Biochem..

[11] Hong J.-Y., Kim G.-H., Kim J.-W., Kwon S.-S., Sato E.F., Cho K.-H., Shim E.B. (2012). Computational modeling of apoptotic signaling pathways induced by cisplatin. BMC Syst. Biol..

[12] Wang R.-S., Saadatpour A., Albert R. (2012). Boolean modeling in systems biology: An overview of methodology and applications. Phys. Biol..

[13] Albert R., Thakar J. (2014). Boolean modeling: A logic-based dynamic approach for understanding signaling and regulatory networks and for making useful predictions. Wiley Interdiscip. Rev. Syst. Biol. Med..

[14] Mackay T.F., Stone E.A., Ayroles J.F. (2009). The genetics of quantitative traits: Challenges and prospects. Nat. Rev. Genet..

[15] Davila-Velderrain J., Martinez-Garcia J.C., Alvarez-Buylla E.R. (2015). Modeling the epigenetic attractors landscape: Toward a post-genomic mechanistic understanding of development. Front. Genet..

[16] Joo J.I., Zhou J.X., Huang S., Cho K.-H. (2018). Determining relative dynamic stability of cell states using boolean network model. Sci. Rep..

[17] An S., Jang S.-Y., Park S.-M., Lee C.-K., Kim H.-M., Cho K.-H. (2023). Global stabilizing control of large-scale biomolecular regulatory networks. Bioinformatics.

[18] Petrenko N., Chereji R.z.V., McClean M.N., Morozov A.V., Broach J.R. (2013). Noise and interlocking signaling pathways promote distinct transcription factor dynamics in response to different stresses. Mol. Biol. Cell.

[19] Ilan Y. (2020). Advanced tailored randomness: A novel approach for improving the efficacy of biological systems. J. Comput. Biol..

[20] Burda Z., Krzywicki A., Martin O.C., Zagorski M. (2011). Motifs emerge from function in model gene regulatory networks. Proc. Natl. Acad. Sci. USA.

[21] Flobak Å., Baudot A., Remy E., Thommesen L., Thieffry D., Kuiper M., Lægreid A. (2015). Discovery of drug synergies in gastric cancer cells predicted by logical modeling. PLoS Comput. Biol..

[22] Chao X., Zao J., Xiao-Yi G., Li-Jun M., Tao S. (2010). Blocking of PI3K/AKT induces apoptosis by its effect on NF-κB activity in gastric carcinoma cell line SGC7901. Biomed. Pharmacother..

[23] Sha M., Ye J., Zhang L.-X., Luan Z.-Y., Chen Y.-B., Huang J.-X. (2014). Celastrol induces apoptosis of gastric cancer cells by miR-21 inhibiting PI3K/Akt-NF-κB signaling pathway. Pharmacology.

[24] Fiedler B., Mochizuki A., Kurosawa G., Saito D. (2013). Dynamics and control at feedback vertex sets. I: Informative and determining nodes in regulatory networks. J. Dyn. Differ. Equ..

[25] Zanudo J.G., Albert R. (2015). Cell fate reprogramming by control of intracellular network dynamics. PLoS Comput. Biol..

[26] Kim Y., Choi S., Shin D., Cho K.-H. (2017). Quantitative evaluation and reversion analysis of the attractor landscapes of an intracellular regulatory network for colorectal cancer. BMC Syst. Biol..

[27] Helikar T., Konvalina J., Heidel J., Rogers J.A. (2008). Emergent decision-making in biological signal transduction networks. Proc. Natl. Acad. Sci. USA.

[28] Kwon Y.-K., Cho K.-H. (2007). Analysis of feedback loops and robustness in network evolution based on Boolean models. BMC Bioinform..

[29] Chisholm R.H., Lorenzi T., Lorz A., Larsen A.K., Almeida L.N.d., Escargueil A., Clairambault J. (2015). Emergence of drug tolerance in cancer cell populations: An evolutionary outcome of selection, nongenetic instability, and stress-induced adaptation. Cancer Res..

[30] Holland S.L., Reader T., Dyer P.S., Avery S.V. (2014). Phenotypic heterogeneity is a selected trait in natural yeast populations subject to environmental stress. Environ. Microbiol..

[31] Wang P., Lü J., Yu X. (2014). Identification of important nodes in directed biological networks: A network motif approach. PLoS ONE.

[32] Mathur R., Rotroff D., Ma J., Shojaie A., Motsinger-Reif A. (2018). Gene set analysis methods: A systematic comparison. BioData Min..

[33] Fumia H.F., Martins M.L. (2013). Boolean network model for cancer pathways: Predicting carcinogenesis and targeted therapy outcomes. PLoS ONE.

[34] Janky R.S., Verfaillie A., Imrichová H., Van de Sande B., Standaert L., Christiaens V., Hulselmans G., Herten K., Naval Sanchez M., Potier D. (2014). iRegulon: From a gene list to a gene regulatory network using large motif and track collections. PLoS Comput. Biol..

[35] Choi M., Shi J., Jung S.H., Chen X., Cho K.-H. (2012). Attractor landscape analysis reveals feedback loops in the p53 network that control the cellular response to DNA damage. Sci. Signal..

[36] Xiong W., Ferrell J.E. (2003). A positive-feedback-based bistable ‘memory module’that governs a cell fate decision. Nature.

[37] Bulusu K.C., Guha R., Mason D.J., Lewis R.P., Muratov E., Motamedi Y.K., Cokol M., Bender A. (2016). Modelling of compound combination effects and applications to efficacy and toxicity: State-of-the-art, challenges and perspectives. Drug Discov. Today.

[38] Jaeger S., Igea A., Arroyo R., Alcalde V., Canovas B., Orozco M., Nebreda A.R., Aloy P. (2017). Quantification of pathway cross-talk reveals novel synergistic drug combinations for breast cancer. Cancer Res..

[39] Barabási A.-L., Gulbahce N., Loscalzo J. (2011). Network medicine: A network-based approach to human disease. Nat. Rev. Genet..

[40] He Q., Xia Z., Lin B. (2016). An efficient approach of attractor calculation for large-scale Boolean gene regulatory networks. J. Theor. Biol..

[41] Yordanov P., Stelling J., Otero-Muras I. (2020). BioSwitch: A tool for the detection of bistability and multi-steady state behaviour in signalling and gene regulatory networks. Bioinformatics.

[42] Gil D.P., Law J.N., Murali T. (2017). The PathLinker app: Connect the dots in protein interaction networks. F1000Research.

[43] Lee D., Cho K.-H. (2018). Topological estimation of signal flow in complex signaling networks. Sci. Rep..

[44] Türei D., Korcsmáros T., Saez-Rodriguez J. (2016). OmniPath: Guidelines and gateway for literature-curated signaling pathway resources. Nat. Methods.

[45] Garcia-Alonso L., Holland C.H., Ibrahim M.M., Turei D., Saez-Rodriguez J. (2019). Benchmark and integration of resources for the estimation of human transcription factor activities. Genome Res..

[46] Patra S., Mohapatra A. (2019). Disjoint motif discovery in biological network using pattern join method. IET Syst. Biol..

