# Supplementary material for: Reducing State Conflicts between Network Motifs Synergistically Enhances Cancer Drug Effects and Overcomes Adaptive Resistance
Source: Cancers (Basel). 2024 Mar 29;16(7):1337. doi: 10.3390/cancers16071337 (PMC11010870; doi:10.3390/cancers16071337)
Supplement: Supplementary file 1 [file cancers-16-01337-s001.zip › cancers-2877579-supplementary.pdf]

Supplementary Materials

Reducing State Conflicts between Network Motifs Synergistically Enhances Cancer Drug Effects and Overcomes Adaptive Resistance

Yunseong Kim, Sea Rom Choi and Kwang-Hyun Cho *

Laboratory for Systems Biology and Bio-Inspired Engineering, Department of Bio and Brain Engineering, Korea Advanced Institute of Science and Technology (KAIST), Daejeon 34141, Republic of Korea; verato@kaist.ac.kr (Y.K.); cgs@biorevert.com (S.R.C.)
* Correspondence: ckh@kaist.ac.kr

(a)

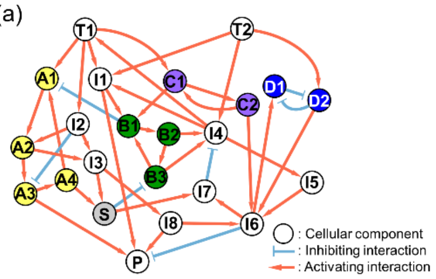

T1 = T1 OR I4
T2 = T2
A1 = (T1 OR A4) AND NOT B1
A2 = A1 OR I2
A3 = A2 OR NOT I2
A4 = A3
B1 = I1 AND C1
B2 = B1
B3 = B2 AND NOT S
C1 = T1 OR C2
C2 = C1
D1 = I6 OR NOT D2
D2 = T2 AND NOT D1
S = A4 OR I3
I1 = T1 OR T2 OR I4
I2 = T1
I3 = A2 AND I2
I4 = T2 OR (B2 AND B3)
I5 = I4
I6 = C2 OR D2 OR I5
I7 = I6
I8 = I3
P = (I1 AND (A3 OR I8)) AND NOT I6

Desired phenotype: an OFF state of the node P

(b)

Green: Desired states; Orange: Undesired states; Grey: ON; White: OFF

Network	Attractor #	T1	T2	A1	A2	A3	A4	B1	B2	B3	C1	C2	D1	D2	S	I1	I2	I3	I4	I5	I6	I7	I8	P
Original	1	0	1	1	1	1	1	1	1	1	1	0	1	1	1	1	1	1	1	1	0	1	1	1
	2	0	1	1	1	1	1	1	1	1	1	0	1	1	1	0	0	1	1	1	0	1	1	0
	3	1	1	1	1	1	1	0	0	0	1	0	0	0	0	0	0	0	0	0	0	1	0	0
	4	1	1	1	1	1	1	0	0	0	1	0	0	0	0	0	0	1	1	0	0	1	1	1
	5	1	1	1	1	0	0	0	1	0	1	0	0	0	0	0	0	1	0	0	0	1	0	1
Regulated	1	0	1	1	1	1	1	0	1	1	1	0	1	1	1	1	1	1	1	1	0	1	1	1
	2	0	1	1	1	1	1	0	1	1	1	0	1	1	1	0	0	1	1	1	0	1	1	0
	3	0	0	1	1	0	0	0	1	1	1	0	0	0	0	0	0	1	1	0	0	1	0	0

Figure S1. The logic equations of exemplary network models.

(a) Logic equations of the exemplary network in Fig. 1a are shown. Cellular components are represented in white circles. Inhibiting and activating interactions are represented in blue blunted and red arrows, respectively. Nodes represented in the same color are within the same group of motifs. **(b)** Identified attractors of the network with asynchronously updating Boolean network modeling scheme.

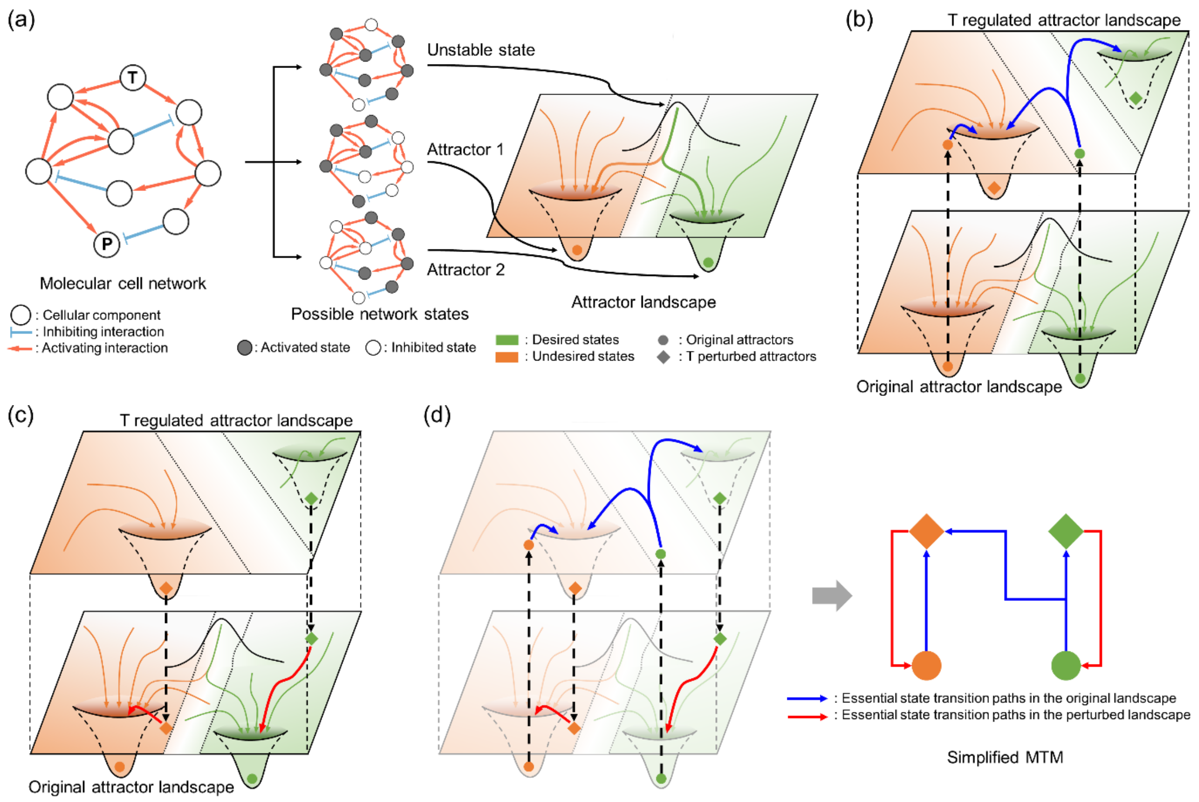

Figure S2. The schematic representation of MTM and random stabilizations.

(a) Illustration of an exemplary molecular cell network that has two attractors and various unstable states. States within the intermediate boundary are colored white which can erratically converge to multiple attractors, causing random stabilizations. **(b)** State transitions are shown after a certain perturbation is induced. After node T is regulated, the attractor landscape changes and the initial states travel to other attractors in neighborhoods, which are depicted with blue arrows. **(c)** Reversal transitions of the node perturbation are shown once the node T is stopped being regulated, or unpinned. The attractor landscape is restored to the original landscape and their network states travel again back to their original landscape, which is depicted with red arrows. **(d)** The attractor-to-attractor transitions, depicted in blue and red arrows from two landscapes, are considered essential dynamics of the exemplary network after regulating node T. The MTM containing essential dynamics of the network can be constructed by merging essential state transitions onto a 2-dimensional path map.

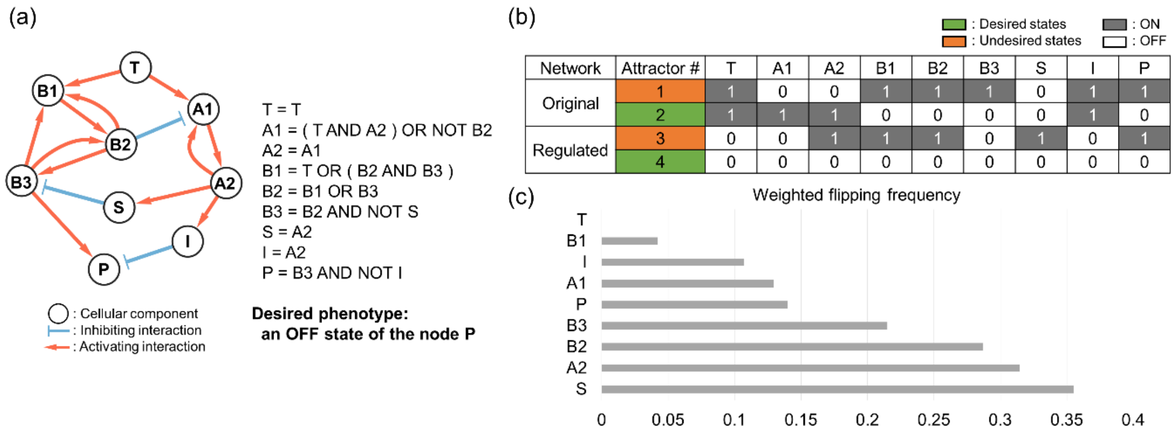

Network	Attractor #	T	A1	A2	B1	B2	B3	S	I	P
Original	1	1	0	0	1	1	1	0	1	1
	2	1	1	1	0	0	0	0	1	0
Regulated	3	0	0	1	1	1	0	1	0	1
	4	0	0	0	0	0	0	0	0	0

Figure S3. The logic equations of exemplary network models.

(a) Logic equations of the exemplary network in Fig. 2a are shown. Cellular components are represented in white circles. Inhibiting and activating interactions are represented in blue blunted and red arrows, respectively. **(b)** Identified attractors of the network with asynchronously updating Boolean network modeling scheme. **(c)** Node S has highest weighted flipping frequency and identified as a synergistic target pair of the node T perturbation with the MTM.

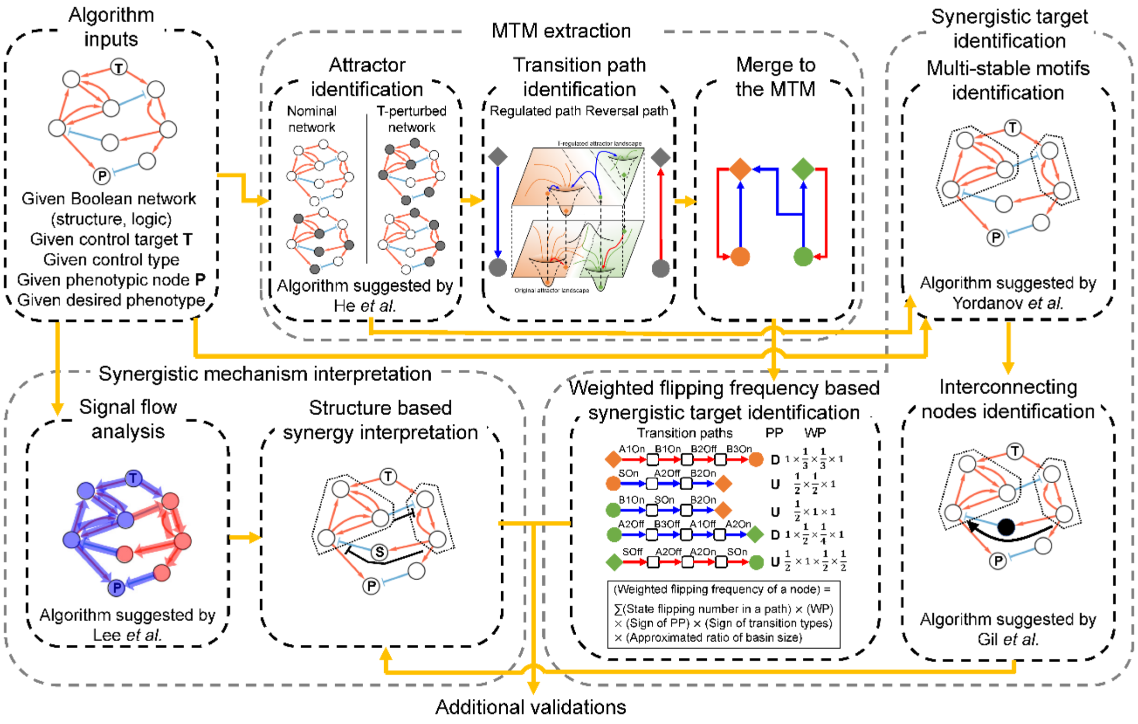

Figure S4. An overall workflow chart of the MTM based synergistic target identification.

With a given Boolean network, control target and desired phenotype, every attractor is identified in both nominal and target node perturbed networks by the algorithm suggested by He *et al.*[1]. The transition paths between every attractor is calculated and merged to the MTM. Also, multi-stable motifs of each attractor are identified by the algorithm suggested by Yordanov *et al.*[2]. Interconnecting links between identified motifs are identified by the algorithm suggested by Gil *et al.*[3]. Weighted flipping frequency of every node is calculated by MTM and the most frequently flipping node is defined and selected as a synergistic target of the given target node. The synergistic mechanism is interpreted by the structural information suggested by Lee *et al.*[4]. The synergism of the combinatorial treatment of the given target and the identified synergistic target is further validated.

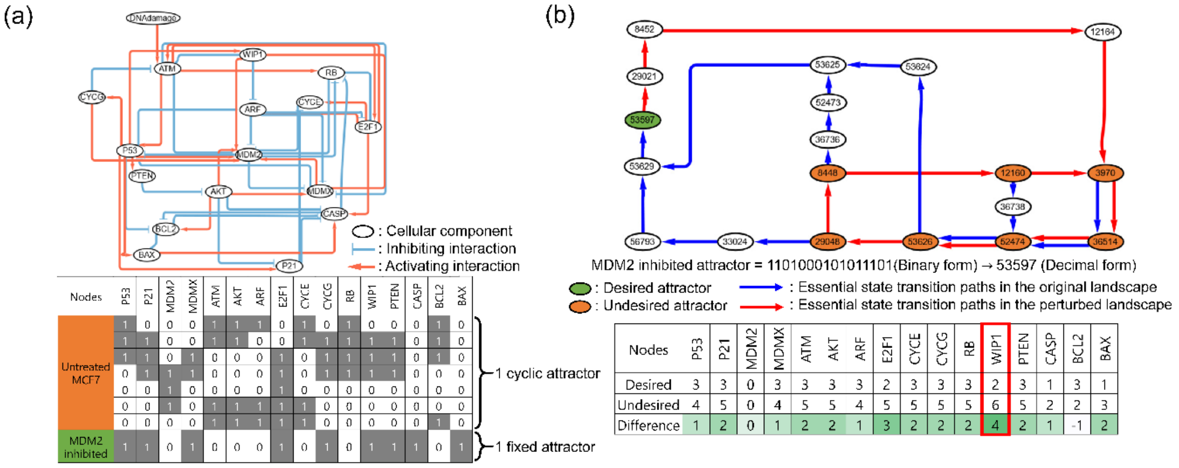

Figure S5. Identification of previously reported synergistic target pairs of the synchronously updated Boolean network model through MTM.

(a) MCF7 cell line network model and its attractors are shown. The MTM is applied on the synchronously updated network model of MCF7 breast cancer cell line constructed by Choi *et al.*[5]. One cyclic attractor of the nominal network model, which is an undesired phenotypic state, and one attractor after inhibition of MDM2 from the network model, which is desired phenotypic state, are shown in the table. **(b)** The MTM of network model with MDM2 inhibition and its suggested synergistic target pair of MDM2 inhibition are shown. For a better representation of each attractor, the attractor states in binary digits are represented in decimal forms. WP is not calculated since the MTM of the Boolean network is deterministic. WIP1 is selected as a synergistic target pair of MDM2 which is the most frequently flipping node during the state transitions after MDM2 inhibition, whereas it flips less during its reversal transitions.

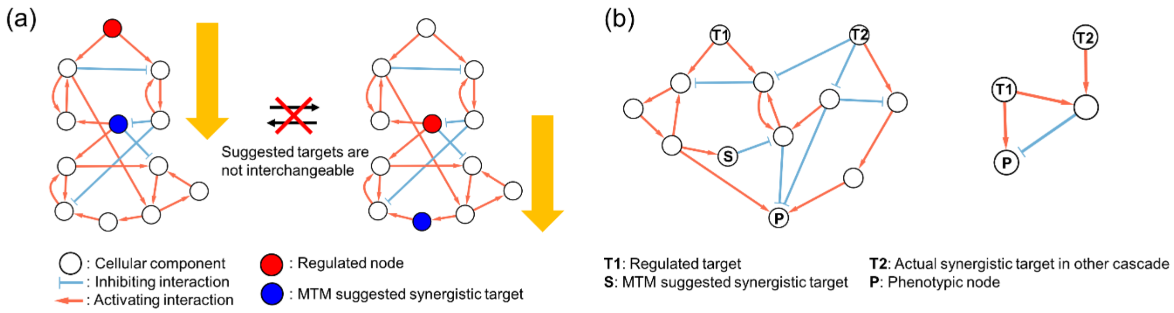

Figure S6. Topological characteristics of synergistic pairs suggested by the MTM.

(a) The MTM extracts perturbation signals that are spread out through hierarchically lower nodes from the regulated node. Thus, the MTM suggests synergistic pairs of the given regulatory target that are located lower which may not interchangeable. **(b)** The characteristics of suggested synergistic targets by the MTM are that it can only identify nodes that are located hierarchically lower from the signaling cascade within a network model or possibly from different signaling cascade if the given regulatory target is directly connected with phenotypic nodes.

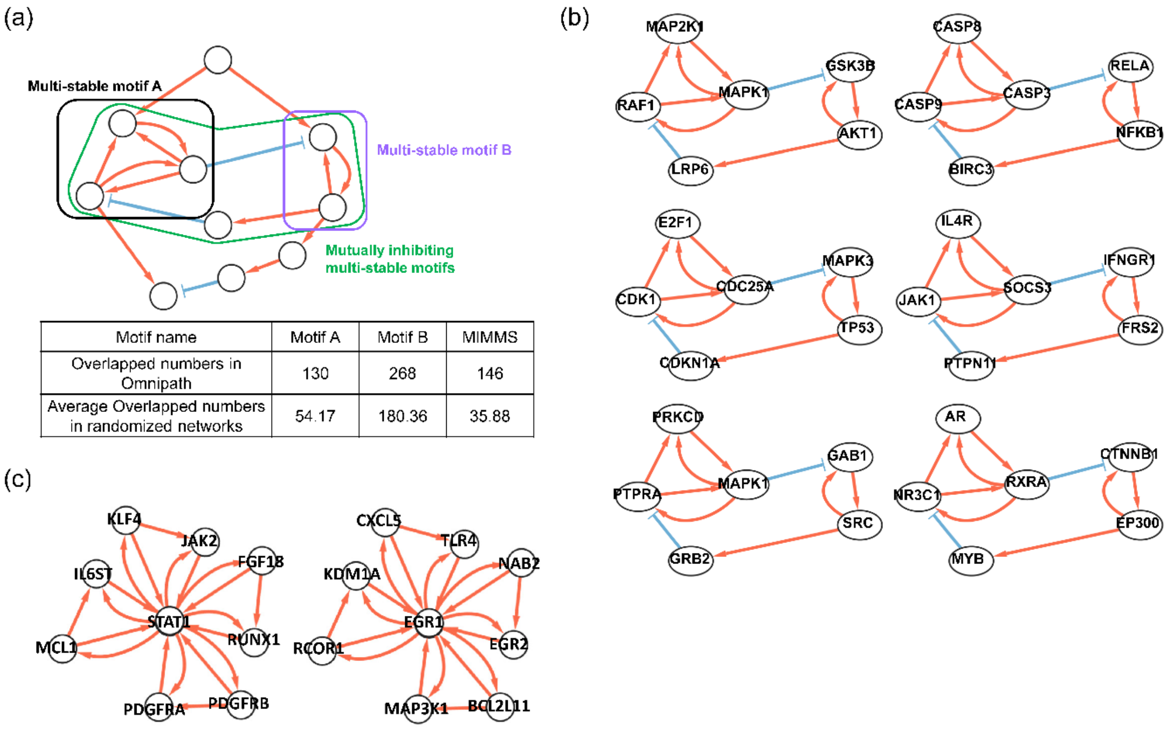

Motif name	Motif A	Motif B	MIMMS
Overlapped numbers in Omnipath	130	268	146
Average Overlapped numbers in randomized networks	54.17	180.36	35.88

Figure S7. Multi-stable motifs and their mutual inhibitions are common in biological network structure.

(a) An exemplary network structure that has three network motifs: multi-stable motif A in a black box, multi-stable motif B in a purple box, and mutually inhibiting structure of multi-stable motif A and B in a green box. The number of overlaps for three motif structures respectively from the given OmniPath[2] curated network structure and randomized Omnipath structures were explored and written in the table. The multi-stable motifs and the mutually inhibiting structure are more frequently occurring in the biological network structure than those of random networks. **(b)** The specific structures in Fig. S5a is not only frequently occurs in the biological network but also closely related with various phenotypic gene sets. **(c)** Beside the mutually inhibiting structures, complex network structures constructed by multi-stable motifs are repeatedly existed in the biological network.

References

1 He, Q., Xia, Z. & Lin, B. An efficient approach of attractor calculation for large-scale Boolean gene regulatory networks. *Journal of Theoretical Biology* **408**, 137-144 (2016).

2 Yordanov, P., Stelling, J. & Otero-Muras, I. BioSwitch: a tool for the detection of bistability and multi-steady state behaviour in signalling and gene regulatory networks. *Bioinformatics* **36**, 1640-1641 (2020).

3 Gil, D. P., Law, J. N. & Murali, T. The PathLinker app: connect the dots in protein interaction networks. *F1000Research* **6** (2017).

4 Lee, D. & Cho, K.-H. Topological estimation of signal flow in complex signaling networks. *Scientific reports* **8**, 5262 (2018).

5 Choi, M., Shi, J., Zhu, Y., Yang, R. & Cho, K.-H. Network dynamics-based cancer panel stratification for systemic prediction of anticancer drug response. *Nature communications* **8**, 1-12 (2017).
